# Transgenic *Drosophila* for Investigating *DUX4* and *FRG1*, Two Genes Associated with Facioscapulohumeral Muscular Dystrophy (FSHD)

**DOI:** 10.1371/journal.pone.0150938

**Published:** 2016-03-04

**Authors:** Takako I. Jones, Megan Parilla, Peter L. Jones

**Affiliations:** 1 The Department of Cell and Developmental Biology, University of Massachusetts Medical School Worcester, Massachusetts, United States of America; 2 The Department of Cell and Developmental Biology, University of Illinois at Urbana-Champaign, Urbana, Illinois, United States of America; INSERM UMR S_910, FRANCE

## Abstract

Facioscapulohumeral muscular dystrophy (FSHD) is typically an adult onset dominant myopathy. Epigenetic changes in the chromosome 4q35 region linked to both forms of FSHD lead to a relaxation of repression and increased somatic expression of DUX4-fl (DUX4-full length), the pathogenic alternative splicing isoform of the *DUX4* gene. DUX4-fl encodes a transcription factor expressed in healthy testis and pluripotent stem cells; however, in FSHD, increased levels of DUX4-fl in myogenic cells lead to aberrant regulation of target genes. DUX4-fl has proven difficult to study *in vivo*; thus, little is known about its normal and pathogenic roles. The endogenous expression of DUX4-fl in FSHD-derived human muscle and myogenic cells is extremely low, exogenous expression of DUX4-fl in somatic cells rapidly induces cytotoxicity, and, due in part to the lack of conservation beyond primate lineages, viable animal models based on DUX4-fl have been difficult to generate. By contrast, the *FRG1* (FSHD region gene 1), which is linked to FSHD, is evolutionarily conserved from invertebrates to humans, and has been studied in several model organisms. FRG1 expression is critical for the development of musculature and vasculature, and overexpression of FRG1 produces a myopathic phenotype, yet the normal and pathological functions of FRG1 are not well understood. Interestingly, *DUX4* and *FRG1* were recently linked when the latter was identified as a direct transcriptional target of DUX4-FL. To better understand the pathways affected in FSHD by DUX4-fl and FRG1, we generated transgenic lines of *Drosophila* expressing either gene under control of the UAS/GAL4 binary system. Utilizing these lines, we generated screenable phenotypes recapitulating certain known consequences of DUX4-fl or FRG1 overexpression. These transgenic *Drosophila* lines provide resources to dissect the pathways affected by DUX4-fl or FRG1 in a genetically tractable organism and may provide insight into both muscle development and pathogenic mechanisms in FSHD.

## Introduction

Facioscapulohumeral muscular dystrophy (FSHD), one of the most prevalent late onset myopathies [~5–12 clinically affected subjects per 100,000 [1, 2]], is characterized by progressive, often asymmetric, weakness and atrophy of specific muscle groups [3–5]. The onset of clinical presentation normally occurs in the second or third decade of life with the muscles of the face and upper body typically affected first, followed by muscles of the lower extremities [3, 4]; however, there is a great range in clinical severity between FSHD subjects. For example, in the severe infantile cases, clinical weakness is apparent from early childhood [6–8], while other carriers may remain asymptomatic throughout their lifetimes [3, 9–15]. Overall, individual FSHD patients exhibit wide variability in age of onset, disease progression, and clinical severity, suggestive of genetic or epigenetic modifiers of the pathogenic pathway [3, 4, 7, 9, 10, 14–17].

**Fig 1:**
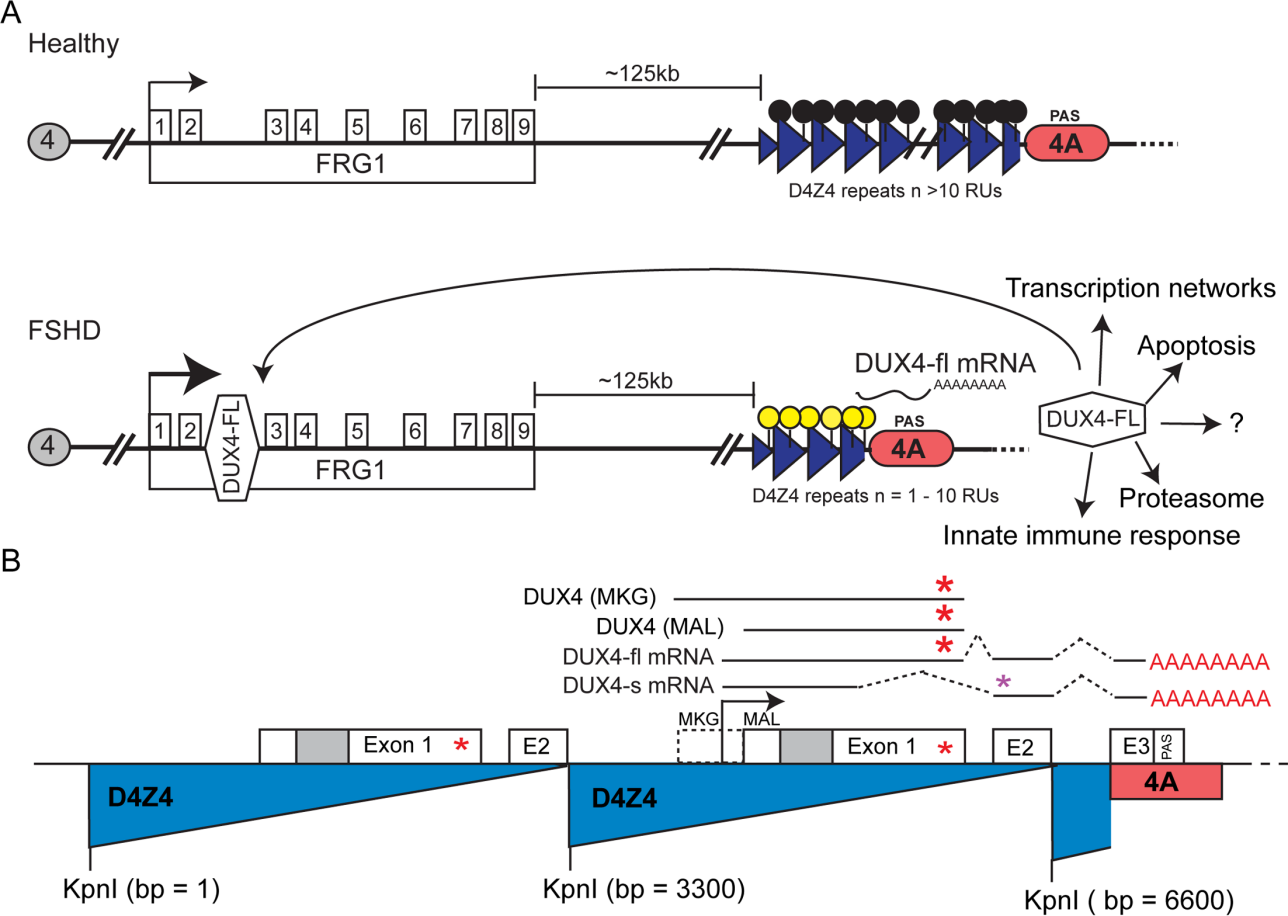
The DUX4 model of FSHD. (A) A model of the human system, which represents a summary of published work relevant to understanding FRG1 and DUX4 in relation to FSHD supplied to aid the reader with context, showing the FSHD-associated human chromosome 4q35 D4Z4 macrosatellite in healthy (upper) and FSHD (lower) subjects. In healthy subjects, the D4Z4 array consists of between 11 and ~120 D4Z4 repeat units (RU) and is epigenetically repressed (black lollipops). FRG1 is ubiquitously expressed, but there is no (or very little) polyadenylated DUX4-fl mRNA expression. In FSHD1 subjects, the D4Z4 array consists of between 1 and 10 D4Z4 RU, is epigenetically derepressed (yellow lollipops), and a significantly higher fraction of cells than in healthy subjects express polyadenlylated DUX4-fl mRNA. The DUX4-FL protein, a transcription factor that regulates many genes, can bind to an enhancer located in intron 2 of *FRG1*, and expression levels of FRG1 are moderately increased in these DUX4-FL expressing cells [34]. (B) The polyadenylated DUX4-fl mRNA is derived from the distal repeat of the D4Z4 array. There are two potential translation start sites for DUX4-fl, termed MKG and MAL. Transgenic *Drosophila UAS-DUX4-fl*^*MKG*^ and *UAS-DUX4-fl*^*MAL*^ contain the coding sequence, codon optimized for *Drosophila*, from the indicated start codon through the stop codon (red *) in exon 1. Please see the following reviews for relevant references relating to FSHD depicted in this figure [17, 21, 24].

There are two genetic classes of FSHD (FSHD1, OMIM 158900; FSHD2, OMIM 158901) that share a common pathogenic mechanism linked to epigenetic changes in the chromosome 4q35 D4Z4 macrosatellite array and subtelomeric region [17–19]. This strong epigenetic component of FSHD may account for much of the incomplete penetrance and high clinical variability in the disease presentation [15–17, 20, 21]. Epigenetic derepression of the 4q35 D4Z4 region leads to the aberrant increased expression of the pathogenic isoform of the *DUX4* gene encoded within the 4q35 D4Z4 array (Fig 1A) [16, 17, 19, 22–24]. Thus, FSHD is essentially a dominant gain-of-function disease, which makes it amenable to being recapitulated, at least in part, by transgenic overexpression in model organisms.

The FSHD-associated *DUX4* gene encodes at least two different protein isoforms generated through alternative mRNA splicing (Fig 1B): a non-pathogenic “short” form of unknown function (DUX4-S) expressed at low levels in healthy somatic cells and a longer “full-length” form (DUX4-FL) that is expressed in the male germ line and can be highly cytotoxic when expressed in somatic cells [23, 25–28]. Only expression of the DUX4-FL isoform is linked to FSHD [14, 19, 23]. DUX4-fl encodes a DNA-binding transcription factor with a paired homeodomain, and DUX4-FL-specific targets include genes expressed in the germ line and in early development, immune mediators, and retroelements (Fig 1A) [29, 30]. These DUX4-FL targets are misregulated in FSHD and, although the mechanisms are still unclear, it is thought that aberrant expression of one or more of these targets ultimately lead to accumulated muscle pathology [29–31].

Interestingly, two proposed alternative FSHD candidate genes, *FRG1* (FSHD region gene 1) [32] and *FRG2* (FSHD region gene 2) [33], localized proximal to the chromosome 4q35 D4Z4 array, were recently identified as direct DUX4-FL target genes [34, 35], thus linking misexpression of these genes to the widely accepted DUX4 model of FSHD [19]. The molecular function of FRG2 remains unknown; no FRG2 protein has ever been reported and overexpression of the *FRG2* mRNA in mice resulted in no apparent phenotype [36]. By contrast, FRG1 is an important for normal development of the vertebrate and invertebrate musculature and vasculature [36–39]; overexpression of FRG1 leads to a severe myopathy in mice, adversely affects muscle development and angiogenesis in *Xenopus*, disrupts muscle structure and organization in *C*. *elegans*, and causes primary defects in myogenic stem cells [27, 36–40]. However, while some studies reported FRG1 misregulation in FSHD [36, 41, 42], many others failed to find upregulation of *FRG1* mRNA or protein in FSHD [43–47]. It is possible that FRG1 is only misexpressed in the minor subset of DUX4-fl expressing FSHD myocytes since DUX4-fl expression can directly influence FRG1 expression, which could account for the difficulty in identifying changes in expression levels in patient biopsies. Thus, in addition to DUX4-fl, misexpression of FRG1 may contribute to FSHD pathology and therefore, FRG1 could be a potential therapeutic target for FSHD.

Little is known about the cellular pathways affected by DUX4-FL or FRG1 expression during early development, myogenesis, or in FSHD. We hypothesized that overexpression models generated in a genetically tractable system would provide insight into the pathways affected by DUX4 and FRG1 through the identification of enhancers and suppressors of their functions. DUX4-FL expression consistently leads to apoptosis in somatic cells and adversely affects fertility in vertebrate systems [27, 28, 48, 49], and the apoptotic pathway is highly conserved from invertebrates to human [50, 51]. Thus, although DUX4 is an old world primate-specific gene, a main pathogenic pathway ultimately activated by DUX4-FL expression is conserved. Similarly, the mechanism by which increased expression of FRG1 leads to myopathy is not understood. However, both the sequence and all known biological functions of FRG1 are highly conserved between invertebrates and human [52], suggesting that the myopathic mechanism is also conserved. *Drosophila* has been used to perform screens for human gene functions by assaying for overexpression phenotypes and the functions of many human genes are highly conserved [53]. In addition, *Drosophila* has been used successfully to model neuromuscular diseases, including six muscular dystrophies, for investigating pathogenic mechanisms and performing genetic and pharmacologic screens [54–63]. Therefore, we generated *Drosophila* transgenic lines that express DUX4-fl or FRG1 under the control of the GAL4-upstream activation sequence (UAS) [64, 65] to serve as tools to investigate the pathways affected by increased FRG1 or DUX4-FL expression. Importantly, expression of these genes in *Drosophila* recapitulated the major phenotypes observed in vertebrate systems, apoptosis for DUX4-FL and disrupted musculature for FRG1. In addition, a set of conditions was identified for both the DUX4-fl and FRG1 lines that produce readily screenable phenotypes for potential use in future screens for genetic enhancers and suppressors.

## Material and Methods

### *Drosophila* strains

The following fly stocks were obtained from Bloomington Stock Center: *DJ667 GAL4* (stock #8171), Act5C-GAL4 (stock #4414), *tubP-GAL4* (stock #5138), *longGMR-GAL4* (stock 8605), *nanos-GAL4*:*VP16* (stock #7312).

### Generation of transgenic lines

A DNA fragment encoding the HA epitope tag (YPYDVPDY) preceded by a start codon was inserted between the *EcoR*I/*Bgl*II sites of the pCaSpeR3 vector [66] to generate pCaSpeR3 HA. The DmFRG1-coding sequence was amplified by PCR from *yw* cDNA using DmFRG1 5’ *Bam*HI and DmFRG1 3’ *Xba*I primers (S3 Table) and then subcloned between the *Bgl*II/*Xba*I sites of the pCaSpeR3 HA vector to generate HA-DmFRG1- pCaSpeR3. This plasmid was used to generate *UAS-DmFRG1* transgenic lines by standard transformation procedures.

The human DUX4-coding sequence was codon optimized for expression in *Drosophila* (http://www.genscript.com/cgi-bin/tools/codon_freq_table), and the entire DUX4-fl^MKG^ coding sequence was synthesized and subcloned into the pUC57 vector, generating HA-DUX4-fl^MKG^ (GenScript USA Inc., S1 Fig). The HA-DUX4-fl^MKG^ fragment was subcloned between the *Not*I/*Xba*I sites of the UASp or pUAST vectors. The shorter HA-DUX4-fl^MAL^ sequence was PCR-amplified from HA-DUX4-fl^MKG^-pUC57 and cloned between the *Not*I/*Xba*I sites of the UASp or pUAST vectors. UASp-HA-DUX4-fl^MKG^, pUAST-HA-DUX4-fl^MKG^, UASp-HA-DUX4-fl^MAL^, and pUAST-HA-DUX4-fl^MAL^ plasmid DNAs were injected by Duke University Model Systems Genomics Core in 2010 (this service is no longer available), and G_0_ larvae (the injectees) were sent to our laboratory for further crosses to establish and balance the lines.

### Production of FRG1 antibodies

Two DmFRG1 rabbit polyclonal antibodies were generated (GenScript Corporation, Piscataway, NJ) against synthesized peptides targeting amino acid sequences 228–241 for DM1 and 247–262 for DM2 (NP_649202), and then affinity purified prior to use.

### Western Blotting

Total protein was extracted from 100 heads/thoraces or embryos in RIPA extraction buffer with protease inhibitors and analyzed (50μg total protein per lane) by SDS-PAGE and western blotting. Both DM1 and DM2 affinity purified antibodies were used at 1:1500 dilution followed by 1:5000 Donkey anti-rabbit-HRP antibody (Roche).

### qRT-PCR

Adult flies with indicated genotypes were collected at 2 days and 10 days after eclosion and kept frozen at -80°C. Each frozen fly was dissected into thorax/legs, abdomen, and head on dry ice, and groups of 40 (20 male and 20 female) were homogenized in TRIzol (Life Technologies) for total RNA extraction as per manufacturer’s instructions. Total RNAs were treated with DNase I on column and purified using the RNeasy mini kit according to the manufacturer’s protocol (QIAGEN). One-step qRT-PCR was performed using 30ng or 15ng of total RNA for DmFRG1 and rp49 expression analysis, respectively (iTaq Universal SYBR Green One-Step Kit, Bio-Rad Laboratories). Cycling conditions were: 50°C for 10 min, 95°C for 1 min, then 40 cycles of 95°C for 10 sec, 60°C for 15 sec, and 72°C for 30 sec. The primers used for qPCR analysis are listed in S3 Table.

### Immunofluorescence

Immunofluorescence of ovaries was performed as described with representative images shown [67]. Eye imaginal discs were dissected from 3rd instar larvae and immunostained as described with representative images shown [68]. Adult thoraxes were dissected and fixed in 4% paraformaldehyde/PBS at 4°C for 1 hour, and then cryoprotected in 12% sucrose/PBS at 4°C overnight before being embedded in OCT (Tissue Tek). Cryosections (10-μm thick) were immunostained as described [69]. Antibodies and dilutions used are: rat anti-HA 3F10 (1:200, Roche), mouse anti-ELAV-9F8A9 (1:10), rabbit anti-DmFRG1 DM1 (1:300), rabbit anti-DmFRG1 DM2 (1:300), and Alexa 594 or 488-conjugated secondary antibodies. Nuclei were visualized with DAPI (Invitrogen). Elav-9F8A9 was deposited to the Developmental Studies Hybridoma Bank by G.M. Rubin. For polytene chromosome spreads, salivary glands were dissected from >100 third-instar larvae grown at 25°C and immunofluorescence was performed as described [70].

### Histology

Newly hatched flies were placed at 29°C incubator for 10 days before fixation in Carnoy’s solution at room temperature for overnight. Whole flies (n = 20) were further processed for paraffin sections as described [71]. Paraffin sections (12 μm-thick) were allowed to dry overnight and stained with hematoxyline and eosin.

### Flight assay

Flies were placed at 29°C incubator for 10 days prior to testing. Flight ability was determined by placing 10-day-old flies in a 20-cm glass vial, allowing the flies to climb the sides, and then tapping the vial to dislodge them from the sides of the vial. Flies were scored as having impaired flight ability if they dropped vertically and hit the bottom of the vial instead of flying to catch the side of the tube.

### Repository

Transgenic lines will be available through the Bloomington *Drosophila* Stock Center at Indiana University (http://flystocks.bio.indiana.edu/).

## Results

### Generation and characterization of *UAS-DUX4* transgenic lines

Each D4Z4 repeat unit (RU) from the human chromosome 4q35 array contains an open reading frame encoding the DUX4 transcription factor; however, due to the polyadenylation signal residing on a third exon located in the subtelomere distal to the D4Z4 array, only the distal D4Z4 RU produces a stable polyadenylated mRNA and is pathogenic when expressed in myogenic cells [19, 23]. The DUX4-FL coding sequence is contained in a single exon with two potential in-frame methionines that could serve as translation initiation codons: the first at base pair (bp) 4912 (termed the MKG start codon in reference to the first 3 amino acids) and the second at bp 5094 (termed the MAL start codon) using the base alignments from the 5’ KpnI site (bp 1) considered the proximal boundary of the first intact D4Z4 of the lamda42 clone [22, 72]. Mapping of capped mRNA transcripts in myogenic cells identified multiple 5’-capped ends of the *DUX4* mRNA located from bps 4941–4962, between MKG and MAL, suggesting that MAL is a functional translation initiation codon *in vivo*, but not precluding that MKG could be utilized under certain conditions or in other cell types [72]. In fact, the MKG translation initiation site has recently been reported to generate functional protein in human mesenchymal stromal cells [73]. Therefore, we synthesized two DNAs, one encoding the DUX4-FL protein starting from MKG (*DUX4-fl*^*MKG*^) and one encoding DUX4-FL from MAL (*DUX4-fl*^MAL^). Both syntheses were codon optimized for expression in *Drosophila* and contained a single HA epitope tag at the amino terminus (S1 Fig). The two sequences were cloned into the pUASp [74] and pUAST [64] vectors for germ line and somatic expression, respectively, and each of the four constructs was injected into 125 embryos and analyzed (S1 Table).

Injections of *pUAST-DUX4-fl*^*MKG*^ and *pUAST-DUX4-fl*^*MAL*^ transgenes produced very few transgenic flies, which may not be surprising since even low levels of DUX4-FL are highly cytotoxic in vertebrate somatic cells [25, 27, 49], and suggests that this key activity of the human DUX4-FL protein is conserved in *Drosophila*. Interestingly, there were differences between the two transgenes; *pUAST-DUX4-fl*^*MAL*^ produced 7 independent transgenic lines, all of which died prior to reproduction, while *pUAST-DUX4-fl*^*MKG*^ produced 8 transformants, 1 of which died and 7 of which were sterile. Thus, no stable transgenic lines were generated from either DUX4-fl sequence using the pUAST somatic expression vector.

*UAS-DUX4-fl* transgenic lines were successfully generated using the UASp germline expression vector, with transformants showing 0% mortality. Considering the lethal phenotype generated with the somatic vector, this was surprising since one might expect low-level leaky expression in somatic cells even with the germline vector. Likely the transgenes in these cases inserted into regions of the fly genome that were less favorable to transcription and suppressed their expression allowing for successful embryogenesis. Again, there were differences found between the two constructs; offspring from both were fertile, however, the *UAS-DUX4-fl*^*MKG*^ transgene produced very weak lines which were ultimately lost, while the *UASp-DUX4-fl*^*MAL*^ transformants were relatively healthy and two independent lines, *UASp-DUX4* #26 (2nd chromosome insertion) and #55 (3rd chromosome insertion) were successfully established and used in the following experiments.

**Fig 2:**
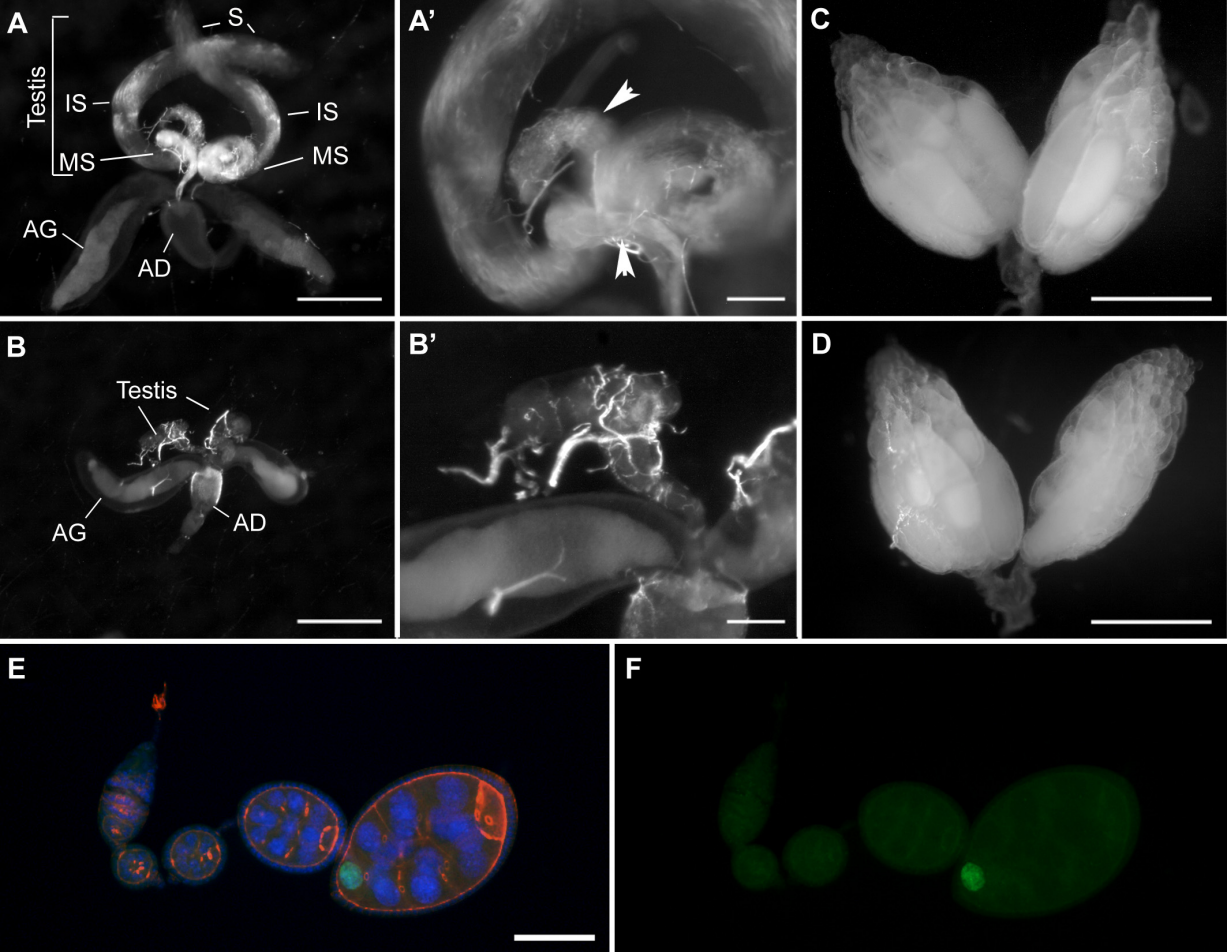
Effects of DUX4-fl expression on the *Drosophila* germline. Adult testis and reproductive tract of *nosGAL4*:*VP16/+* (A and A’) and *nosGAL4*:*VP16/+*, *UASp-DUX4/+* (B and B’). The *nosGAL4*:*VP16/+* has normally developed testis showing spermatagonia (S) progressing to developing immature sperm (IS) and accumulated mature sperm (MS). The *nosGAL4*:*VP16*, *UASp-DUX4* male has an extremely small testis with relatively normal accessory gland (AG) and anterior ejaculatory duct (AD). Magnified picture of *nosGAL4*:*VP16*, *UASp-DUX4* testis (B’) shows lack of fully matured sperm that are observed in normal testis (A’, white arrowheads). Adult ovaries of the *nosGAL4*:*VP16/+* (C) and *nosGAL4*:*VP16/+*, *UASp-DUX4/+* (D). The *nosGAL4*:*VP16/+*, *UASp-DUX4/+* ovaries are filled with developing oocytes and are indistinguishable from normal ovaries (C). E and F) Immunostaining of ovariole from *nosGAL4*:*VP16/+*, *UASp-DUX4/+* shows HA-DUX4-FL (green) is expressed in mid stage of oogenesis (stage 8); DAPI (blue) and phalloidin (red) staining are shown in the overlay. Bar = 500 μm in panels A-D, 100 μm in panels A’ and B’, and 50 μm in panel E.

In humans, DUX4-fl is expressed in the male germline of healthy individuals, while expression in the female germline has not been investigated. To determine the effect of DUX4-fl expression on the *Drosophila* male and female germlines, *UASp-DUX4* flies were crossed with *nanos (nos) GAL4*:*VP16*, and the testes and ovaries were examined. Neither male nor female *nosGAL4*:*VP16; UASp-DUX4* showed lethality, however, all male *nosGAL4*:*VP16; UASp-DUX4* flies were sterile and had malformed testis (Fig 2 and Table 1). In contrast, female *nosGAL4*:*VP16; UASp-DUX4* flies were fertile and had apparently normal ovaries. Immunostaining confirmed expression of DUX4-FL during oogenesis. We conclude that DUX4-fl expression has sex-specific effects on the *Drosophila* germline with no adverse effects on the ovaries or female fertility, while the *Drosophila* testis, as opposed to the case of human males, cannot tolerate DUX4-fl expression.

**Table 1:** DUX4 expression phenotypes.

GAL4 Driver	Male	Female	Phenotype
**Germline Expression:** *nosGAL4*:*VP16/nosGAL4*:*VP16* x *UASp-DUX4/Balancer*			
*nosGAL4*:*VP16/+; CyO/+*	64	87	NA
*nosGAL4*:*VP16/+; UASp-DUX4 #26/+*	61	74	Male Sterility
*nosGAL4*:*VP16/+; TM3/+*	53	78	NA
*nosGAL4*:*VP16/+; UASp-DUX4 #55/+*	68	86	Male Sterility
**Ubiquitous Expression:** *tubP-GAL4/TM3* x *UASp-DUX4/Balancer*			
*CyO/+; tubP-GAL4/+* or *UASp-DUX4 #26/+; TM3/+* or *CyO/+; TM3/+*	137	149	NA
*UAS-DUX4 #26/+*; *tubP-GAL4/+*	0	0	Lethal
*tubP-GAL4/TM3* or *UASp-DUX4 55/TM3*	129	138	NA
*tubP-GAL4 / UASp-DUX4 #55*	0	0	Lethal
**Ubiquitous Expression:** *Act5C-GAL4/TM3* x *UASp-DUX4/Balancer*			
*CyO/+; Act5C-GAL4/+* or *UASp-DUX4 #26/+; TM3/+* or *CyO/+; TM3/+*	50	58	NA
*UASp-DUX4 #26/+; Act5C-GAL4*/+	0	0	Lethal
*Act5C-GAL4/TM3* or *UASp-DUX4 #55/TM3*	103	110	NA
*Act5C-GAL4* / *UASp-DUX4 #55*	0	0	Lethal
**Adult Muscle Expression:** *DJ667 GAL4/TM3* x *UASp-DUX4/Balancer*			
*CyO/+; DJ667 GAL4/+* or *UASp-DUX4 #26/+; TM3/+* or *CyO/+; TM3/+*	81	74	NA
*UASp-DUX4 #26/+; DJ667 GAL4/+*	0	0	Lethal
*DJ667 GAL4/TM3* or *UASp-DUX4 #55/TM3*	88	91	NA
*DJ667 GAL4 / UASp-DUX4 #55*	0	0	Lethal
**Eye Expression:** *lGMR-GAL4/lGMR-GAL4* x *UASp-DUX4/Balancer*			
*lGMR-GAL4/CyO*	58	84	NA
*lGMR-GAL4 / UASp-DUX4 #26*	1	4	Pupal Lethal; Eye Phenotype
*lGMR-GAL4/+; TM3/+*	31	41	NA
*lGMR-GAL4/+*; *UASp-DUX4 #55/+*	0	27	Pupal Lethal; Eye Phenotype

The numbers of male and female adult flies with the indicated genotype are listed along with any aberrant phenotype. NA indicates no discernible phenotype.

### A *Drosophila* model of DUX4-fl for identifying genetic interactions

**Fig 3:**
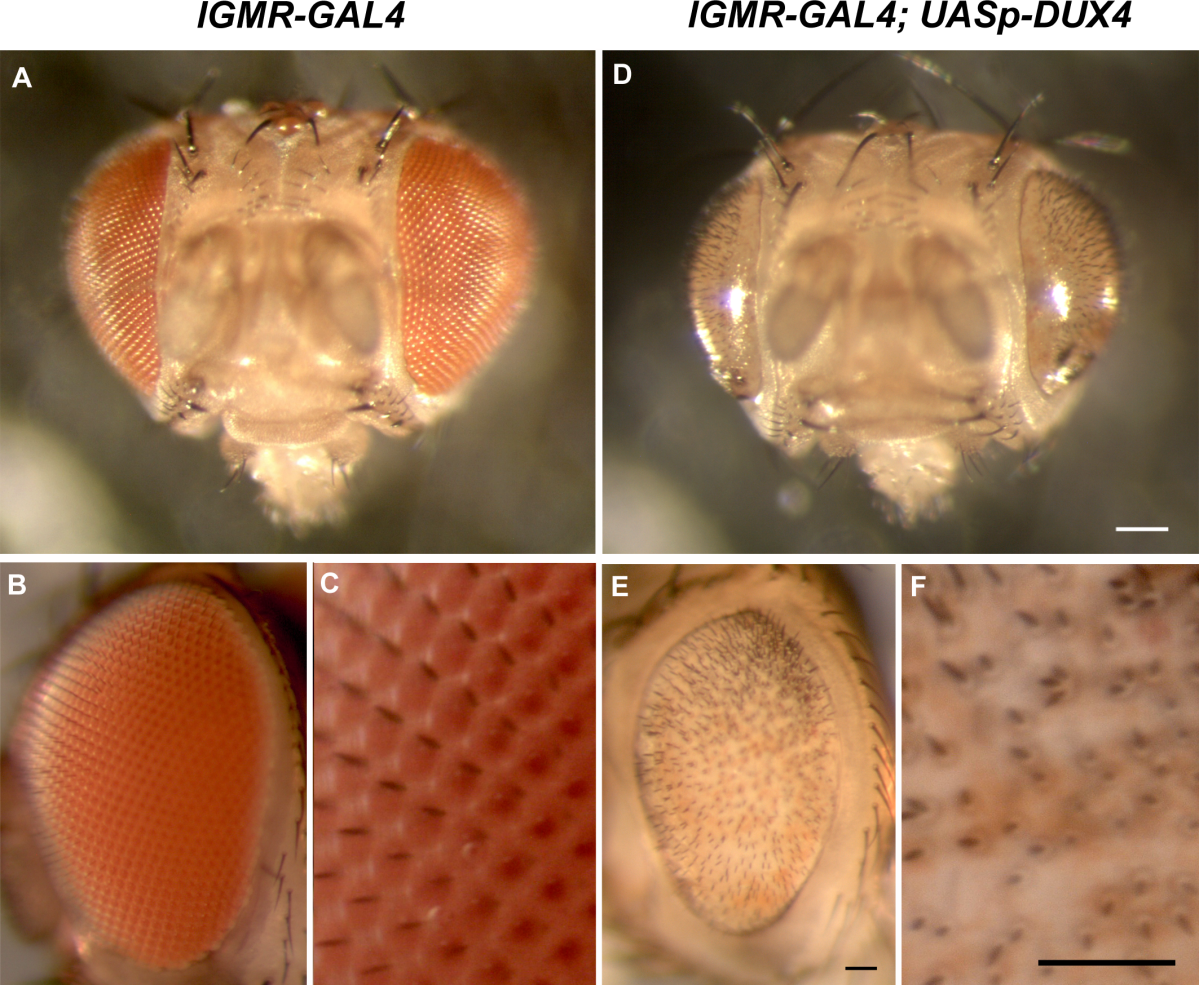
Ectopic *DUX4-fl* expression by *lGMR-GAL4* produces a readily screenable eye phenotype. A-C) Compound eyes of *lGMR-GAL4* consist of precisely organized and pigmented ommatidia and bristles. D-F) Eyes of *lGMR-GAL4/+*, *UASp-DUX4/+* are the usual size but lack organized ommatidia. Corneal lenses and pigment cells are completely missing; bristles are formed, but irregularly dispersed. Bar = 100 μm in panel D; Bar = 50 μm in panels E and F.

**Fig 4:**
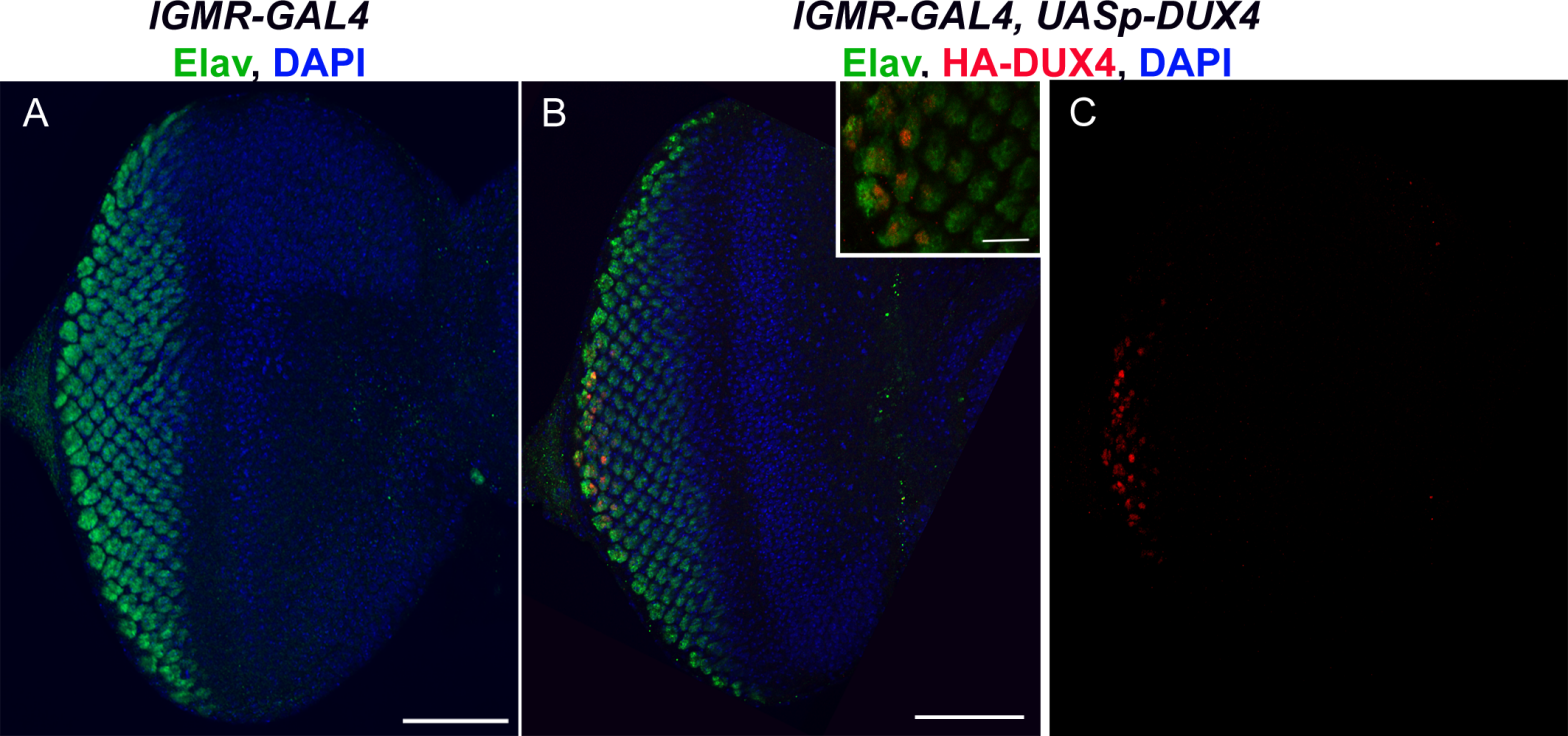
Expression of DUX4-FL in eye imaginal disc leads to disruption of eye formation. Third instar eye imaginal discs from A) *lGMR-GAL4/+* or B and C) *lGMR-GAL4/+; UASp-DUX4/+* were immunostained for the photoreceptor marker ELAV (green) or HA-DUX4 (red), and stained for DAPI (blue). C) Expression of DUX4-FL protein was detected in mature photoreceptors posterior to the morphogenetic furrow. Bar = 50 μm and 10 μm in the magnified picture.

DUX4-fl is highly cytotoxic when expressed in vertebrate somatic cells in culture or during vertebrate embryonic development [25, 27, 28, 75]. Ubiquitous expression of DUX4-fl during development induced by *tubulin 1∂ promoter-GAL4* (*tubP-GAL4*) [76] or *Actin5C promoter-GAL4* (*Act5C-GAL4*) resulted in 100% lethality (Table 1) indicating that this developmental lethal effect is conserved in *Drosophila*. We attempted to bypass the developmental lethality by inducing DUX4-fl expression at later stages in the adult muscle by using the *DJ667 GAL4* line reported to express primarily in adult thoracic muscle [77], however, this cross also resulted in 100% lethality (Table 1). Since we were unable to generate viable flies that expressed DUX4-fl during development or specifically in muscle, we attempted to restrict DUX4 expression to the *Drosophila* eye, a commonly used model for developmental processes including cell proliferation, cell signaling, and apoptosis [51, 78–80] as well as for studying transcription factors [81–84]. We crossed *UAS-DUX4* lines with the *long GMR (lGMR)-GAL4* driver line, which expresses GAL4 in the eye under control of five copies of a 38bp *glass*-binding site with higher specificity and less robustness than regular GMR-GAL4 that utilizes a shorter *glass*-binding site [78, 85, 86]. Although lGMR-GAL4 induces transgene expression primarily in the photoreceptors in the eye imaginal disc during third instar larval stage, F1 offspring showed a predominant pupal lethal phenotype (Table 1); the cross between *lGMR-Gal4/ lGMR-Gal4* and *UASp-DUX4 #26/CyO* produced only 5 *lGMR-Gal4/ UASp-DUX4 #26* flies while 142 of *lGMR-Gal4/CyO* were produced. This is likely due to low ectopic expression of DUX4-fl by lGMR-GAL4 in other tissues [87]. Lethality was more pronounced in the F1 males, however, a number of F1 females were born (Table 1) that displayed a readily apparent eye phenotype with 100% penetrance (Fig 3). Immunostaining confirmed DUX4 protein expression in the eye imaginal discs of *GMR-GAL4*, *UAS-DUX4* third instar larvae (Fig 4). Importantly, this DUX4-mediated eye phenotype was distinctly different from those produced by similarly overexpressing other transcription factors in the eye indicating this phenotype was not merely the result of a non-specific effect due to the overexpression of a transcription factor or signaling protein [80–84]. We conclude that we have successfully generated a line of transgenic DUX4-fl *Drosophila* that can be crossed to generate a model that can be utilized for genetic screening to identify potential genetic enhancers and suppressors of DUX4-FL function.

### Generation and characterization of *Drosophila* transgenic lines of *UAS-DmFRG1*

**Fig 5:**
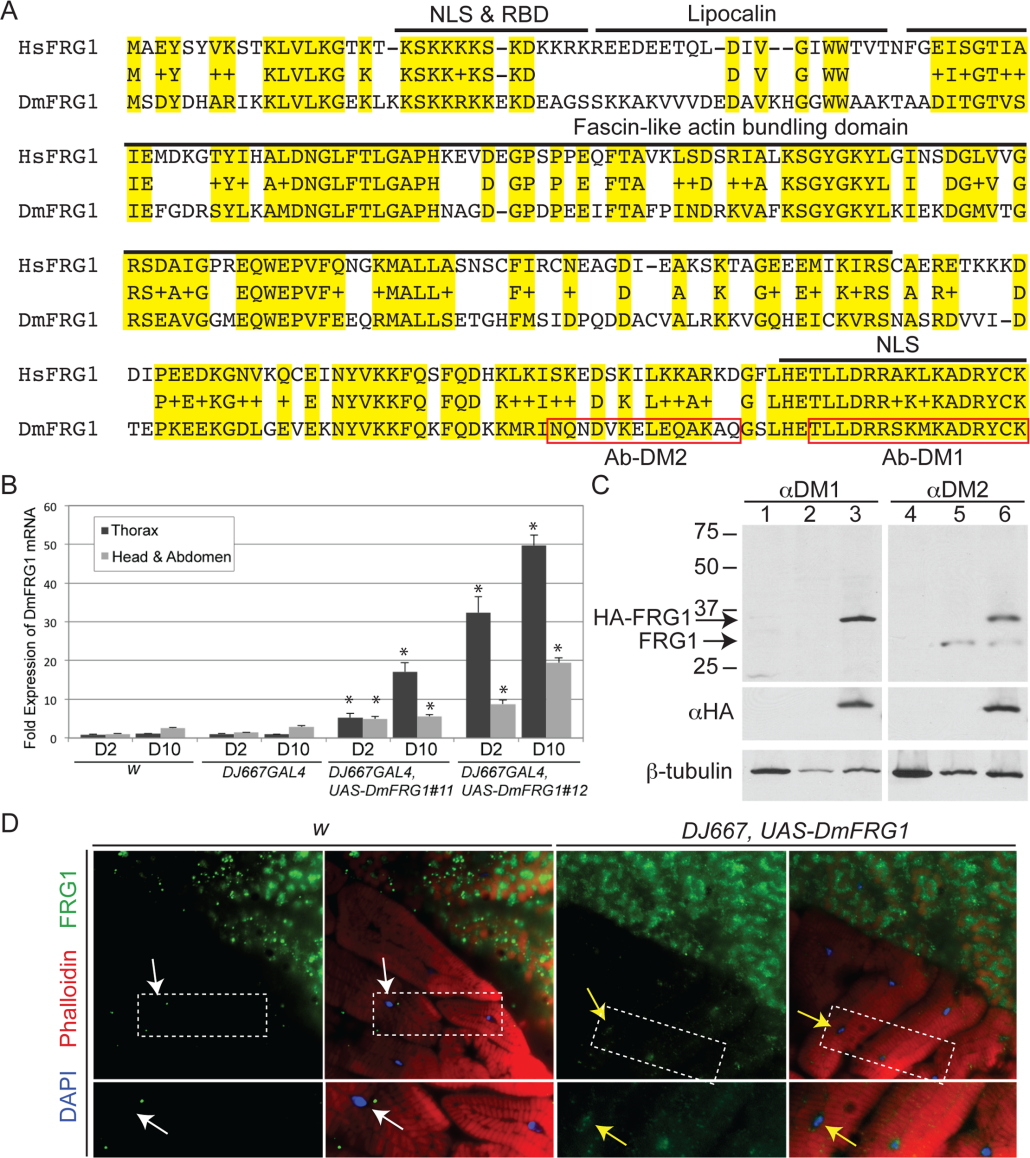
FRG1 is highly conserved between human and *Drosophila*. (A) Alignment of the predicted amino acid sequence for the human (Hs) and *D*. *melanogaster* (Dm) FRG1 orthologs. Conserved amino acids are highlighted in yellow with identical amino acids indicated by the letter and similar amino acids indicated by a +. The functional domains, including the nuclear localization signals (NLS) and RNA binding domain (RBD), are indicated. The peptides used as antigens for generating the DM1 and DM2 antibodies are boxed in red. (B) DmFRG1 expression was assayed by qRT-PCR at day 2 (D2) and day 10 (D10) in the thorax or head and abdomen combined, as indicated. Expression was normalized to *rp49* RNA levels and presented as fold expression compared to D2 or D10 thorax. Induction of DmFRG1 mRNA was significant (* p < 0.01 by Student’s t-test) for both lines when compared with *w* and *DJ667GAL4* alone. (C) Left panel: Western blots of protein extracts from 0–17h embryos (lane 1 and 4) or head/thorax of adult flies (lanes 2, 3, 5, and 6) of *yw* strain (lanes 1, 2, 4, and 5) or *tubP-GAL4*,*UAS-DmFRG1* flies (lanes 3 and 6), probed with the DM1 or DM2 antibody as indicated. Beta-tubulin was used as a loading control. (D) Thoracic muscle tissue immunostained for DmFRG1 (green) and counterstained with DAPI (blue) and phalloidin (red). Endogenous DmFRG1 is not detectable in the nuclei of *yw* flies (white arrows) and accumulates in nuclei when overexpressed (yellow arrows). Zoomed regions displayed in the lower panels are outlined in the upper panels by dotted white boxes.

In humans, aberrant upregulation of FRG1 expression, via epigenetic dysregulation of the FSHD-associated chromosome 4q35 region and, as recently discovered, through the binding of DUX4-FL, has long been a proposed disease mechanism for FSHD [36, 41, 88]. Overexpression of FRG1 or its orthologs in mouse, *Xenopus*, and *C*. *elegans* inhibits myogenic cell migration, leads to defects in myogenic stem cells, disrupts muscle development and function, and recapitulates many features of FSHD [36–40], however the specific pathways leading to these phenotypes are not known. In contrast to primate-specific *DUX4*, human *FRG1* at chromosome 4q35 is highly conserved in metazoans, including *Drosophila*, across its entire open reading frame [32, 52, 89]. *Drosophila FRG1* (*DmFRG1*; *CG6480*) exhibits 50% amino acid identity (66% similarity) with its human ortholog and contains similar functional domains (Fig 5A); therefore, we hypothesized that the *Drosophila* and human FRG1 proteins likely have a conserved biological function and *Drosophila* would be a suitable system for identifying conserved FRG1 affected pathways.

We aimed to use *Drosophila* to create a genetic tool that could be used to identify and better understand the cellular pathways disrupted by FRG1 overexpression, and which therefore might play roles in FSHD pathogenesis. Transgenic *Drosophila* lines containing the coding sequence of *DmFRG1* with an HA epitope tag at the amino terminus were generated using the pUAST vector [64]. Since FSHD predominantly affects adult muscles and the human *FRG1* gene is a direct transcriptional target of the DUX4-FL protein in FSHD muscle [34], the *UAS-DmFRG1* flies were crossed with the *DJ667 GAL4* line, which expresses GAL4 predominantly in adult thoracic muscle [77]. Our qRT-PCR analysis confirmed that *DJ667 GAL4*, *DmFRG1* flies showed increased *FRG1* expression predominantly in thoracic muscle as they aged (Fig 5B). With *UAS-DmFRG1* line 11, there was a 5-fold overexpression of *DmFRG1* mRNA 2 days after hatching that increased to 17-fold at 10 days; with line 12, expression increased from 32-fold to 50-fold. Both double transgenic lines were viable and fertile.

**Fig 6:**
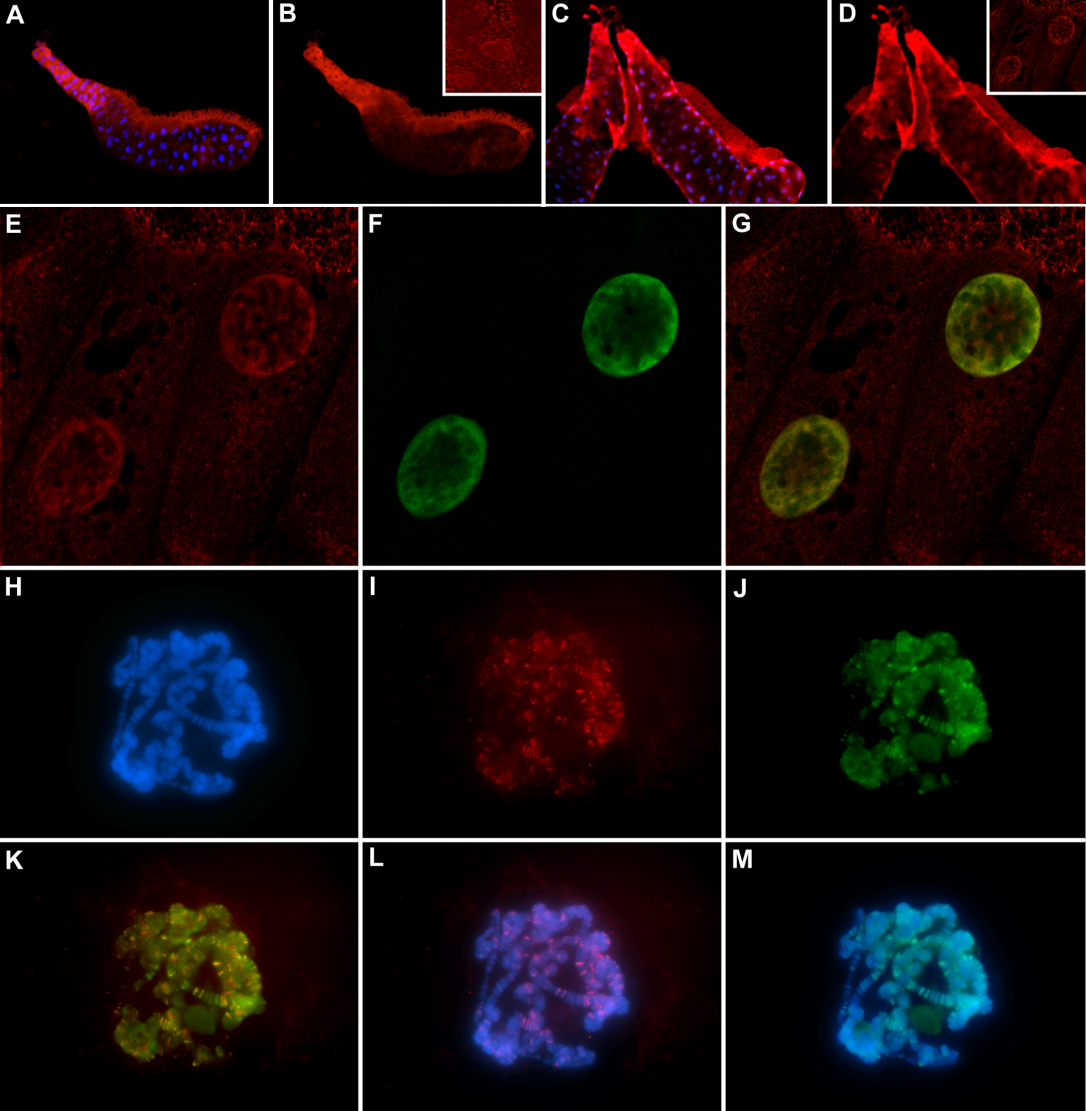
Nuclear localization of endogenous and overexpressed DmFRG1 is conserved. Salivary gland tissue (A-G) and polytene chromosome spreads (H-M) from *w* (A, B) and *tubP-GAL4*, *UAS-DmFRG1* were immunostained for total DmFRG1 using the DM2 antibody (red; A-E, G, I, K, L) or specifically overexpressed DmFRG1 using the HA antibody (green; F, G, J, K, M), and stained with DAPI (blue) to show the nuclei (A, C) or chromosomes (H, L, M). Endogenous DmFRG1 is predominantly cytoplasmic (A, B), while overexpressed DmFRG1 accumulates in the nucleus (E, F, G). In the nucleus, the endogenous and overexpressed DmFRG1 localizations mostly overlap (yellow; G, K).

Tissues from *yw* and *UAS-DmFRG1* adult flies were also assayed for FRG1 protein expression using two affinity-purified polyclonal antibodies (DM-1 and DM-2) generated against the carboxyl terminus of DmFRG1 (Fig 5A). These antibodies detect both the endogenous DmFRG1 protein and the transgenic overexpressed protein (Fig 5C). The transgenic DmFRG1 has an amino terminal HA epitope tag that allows differential detection of endogenous and overexpressed DmFRG1 (Fig 5C). In vertebrates, FRG1 has multiple distinct subcellular localizations: a nuclear pool associated with nascent mRNA transcripts on the chromatin and nucleoli, and a cytoplasmic protein pool [90, 91]. Interestingly, in all model systems we tested previously, endogenous FRG1 was predominantly, but not exclusively, cytoplasmic and the overexpression of FRG1 led to preferential accumulation in the nucleus, and particularly in the nucleolus, suggesting that FRG1 protein levels affect subcellular localization and are likely to be tightly regulated [90, 91]. Immunostaining for DmFRG1 in *Drosophila* muscle and salivary tissues showed a similar pattern (Figs 5D and 6A–6G). As with overexpression in vertebrate cell culture, all detectable DmFRG1 expressed from the transgene was nuclear in *tubP-GAL4*, *UAS-DmFRG1* salivary glands (Fig 6, compare panels E and F). In the nuclear pool of DmFRG1, the overexpressed and endogenous proteins had similar, overlapping subnuclear localization profiles (Fig 6G). The FRG1 associated with polytene chromosomes preferentially localized to the DAPI-poor euchromatic puffs (Fig 6L and 6M), and the overexpressed DmFRG1 showed overlapping localization with the endogenous chromatin-associated DmFRG1 (Fig 6K). Overall, based on this preliminary examination, DmFRG1 behaves similar to its vertebrate orthologs.

### Overexpression of DmFRG1 in adult muscle disrupts organization of the musculature and impairs muscle function

FSHD is typically an adult onset progressive myopathy leading to decreased skeletal muscle mass and ultimately loss of muscle function. FRG1 expression is moderately induced by the DUX4-FL transcription factor in human myocytes due to a human specific DUX4-FL responsive intronic enhancer [92], and overexpression of FRG1 in muscle results in an FSHD-like myopathy in mice and impaired muscle development, function, and movement in *Xenopus* and *C*. *elegans* [36–38, 93]. Therefore, we generated flies overexpressing *DmFRG1* in the adult musculature and assayed them for a basic muscle function: the ability to fly (S1 Movie 1 and 2). The parent lines *DJ667 GAL4* and *UAS-DmFRG1*, which express wild-type levels of *DmFRG1*, are capable of movement and flight indistinguishable from *yw* flies. However, their progeny overexpressing *DmFRG1* in adult thoracic muscles have substantially impaired flight ability (Summarized in S2 Table). These *DJ667 GAL4*, *UAS-DmFRG1* flies have fully developed wings, are capable of walking normally and jumping for takeoff, but were nonetheless incapable of sustained flight (S2 Movie). Thus, overexpression of *DmFRG1* in flight muscles resulted in impaired function.

**Fig 7:**
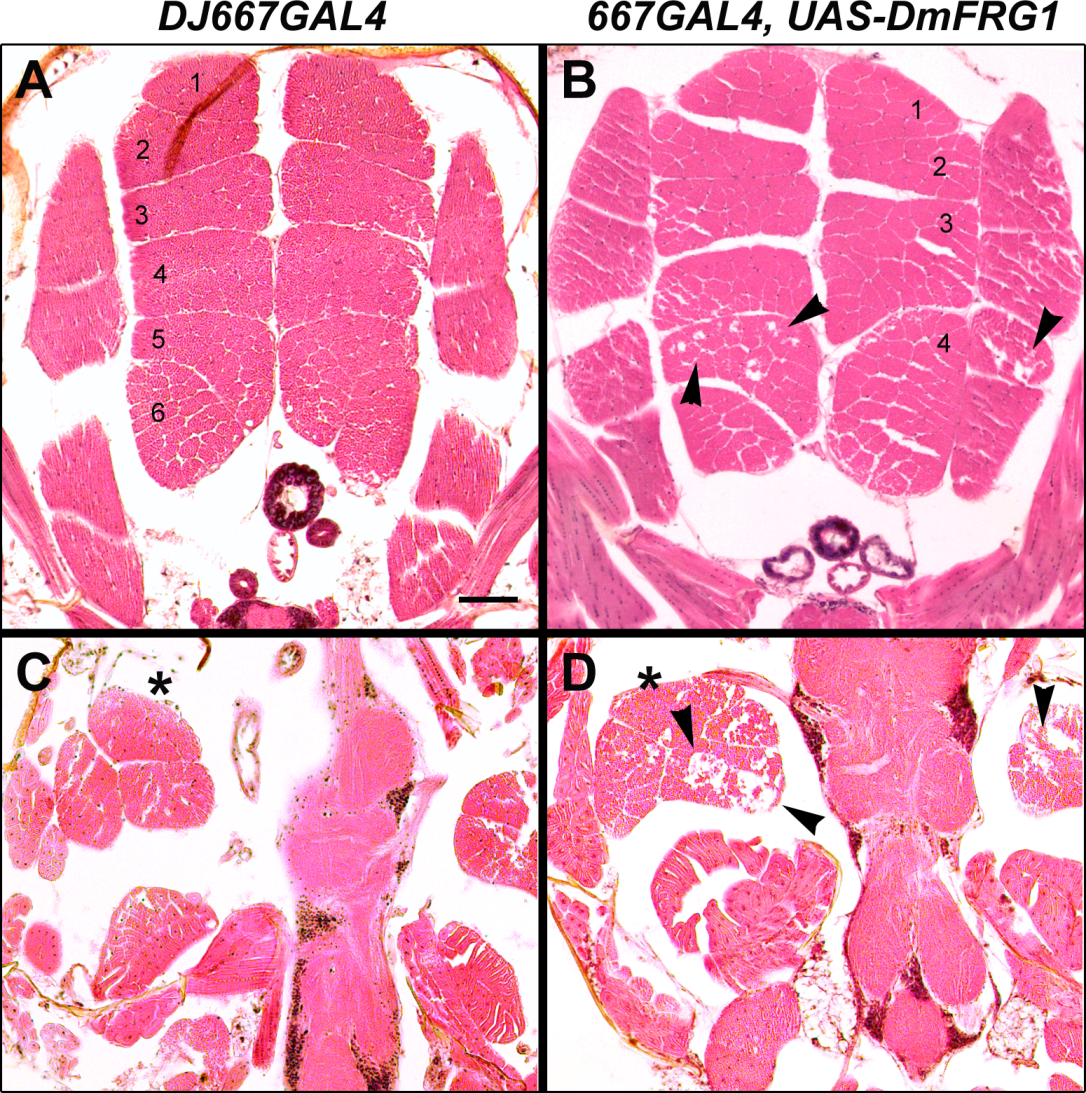
Abnormal musculature in DM-FRG1 overexpressing flies. H&E stained paraffin sections of thoraces from *DJ667GAL4* (A and C) and of *DJ667GAL4; DmFRG1* (B and D) adult flies. (B) Overexpression of DmFRG1 leads to disorganized dorsal longitudinal muscles (DLMs, indirect wing depressors) exhibiting variable numbers and shapes of muscles suggestive of muscle degeneration (arrowheads) compared with the highly organized musculature observed in the controls (A), which exhibit the characteristic 6 distinct DLMs on each side. (C and D) H&E stained paraffin sections of the dorso-ventral tergosternal muscle bundles (indirect wing levators) show histological features characteristic of muscle degeneration (arrowheads). * denotes the same muscle in these flies for a reference. Bar = 500 μm.

Overexpression of FRG1 disrupts the organization and integrity of the musculature in other model organisms and muscle from FRG1 overexpressing mice show features of muscular dystrophy [36–38]. Thus, the thoracic muscles of DmFRG1 overexpressing flies were analyzed histologically and compared with controls for similar indications (Fig 7). Hematoxylin and eosin (H&E) staining of thoraces revealed that DmFRG1 overexpression leads to a disorganized musculature. The dorsal longitudinal muscles (DLMs) in control flies exhibited the typical pattern of 12 organized bundles, six on each side (Fig 7A), however, DLMs from the DmFRG1 overexpressing flies were misshapen, fused together, and were overall highly disorganized (Fig 7B) [58]. Analysis of the dorso-ventral muscle bundles suggested muscle degeneration in the tergosternal muscles (indirect with levator muscles) (Fig 7D). Overall, overexpression of DmFRG1 disrupts the integrity of the musculature and impairs muscle function. We conclude that we have successfully generated a model of *FRG1*-mediated muscle dysfunction that is readily amenable to screening for modifiers of the phenotype.

## Discussion

The FSHD pathogenic mechanisms downstream of *DUX4-fl* misexpression are not well understood due in large part to a lack of phenotypic *DUX4*-based FSHD-like animal models. Circumventing DUX4-fl cytotoxicity and lethality during early development has been a major hurdle inhibiting the generation of viable transgenic FSHD models since even trace amount of spurious DUX4-fl transcription during embryogenesis can be lethal. In fact, generating the only viable transgenic mouse line that expresses DUX4-fl mRNA, the D4Z4-2.5 mice, required more than 450 injections to obtain one viable and fertile line [94], mirroring the situation we faced while generating the *Drosophila DUX4* lines. Compounding the problem, the *DUX4* gene evolved relatively recently and is specific to old-world primates. *DUX4* originated from a gene conversion event in the mammalian *DUXC* macrosatellite array that occurred in the primate and Afrotheria lineages, and subsequently translocated to chromosome 4qter in primates [95, 96]. Therefore, despite apparent homology to ancestral paired homeodomain transcription factors, which is restricted to the DNA binding domains, traditional model systems such as mice, rats, flies, and zebrafish do not have true parental *DUX4* orthologs and no natural models exist [95] and one must always consider potential nonspecific effects when working with DUX4 expressed in non-human systems.

Although generally silent in adult somatic tissues, *DUX4-fl* is expressed normally in human testis, pluripotent stem cells [23], and many somatic tissues of FSHD1 and healthy fetuses, suggesting that the *DUX4* retrogene has likely evolved to function specifically during primate germ line and embryonic development [97, 98]. Presumably, the DUX4-FL protein co-opted the host primate genome and cellular machinery to regulate certain endogenous primate genes and cellular pathways [29–31]. Thus, despite *DUX4* being primate-specific, most of the DUX4-FL target genes and affected pathways are evolutionarily conserved and present in model organisms, allowing introduction of DUX4-FL to produce a potentially useful model of certain aspects of FSHD. In fact, *Xenopus* and zebrafish embryos injected with *DUX4-fl* mRNA and mice expressing *DUX4-fl* via viral infection or transgene expression exhibit phenotypes consistent with FSHD [27, 28, 75, 99]. One caveat is that although many protein-coding DUX4-FL target genes are conserved, their regulatory elements may not be conserved, resulting in gene expression patterns that do not fully overlap with FSHD transcription profiles or those of human cells expressing exogenous *DUX4-fl* [29, 31, 100]. For example, the human *FRG1* gene contains a functional intronic DUX4-FL binding site but this site is absent from the murine *Frg1* gene, rendering it non-inducible by DUX4-FL [92].

A key part of DUX4 evolution in primates includes the silencing of *DUX4-fl* expression in adult somatic tissues, where its expression is deleterious. Upon loss of epigenetic silencing in FSHD, abnormal *DUX4-fl* expression in adult skeletal muscle has pathological consequences by aberrantly activating target genes and *DUX4*-regulated cellular pathways [31]. Low levels of DUX4-FL are highly cytotoxic when expressed in somatic cells and DUX4-FL expression disrupts vertebrate development [25, 27, 28, 49, 75]. This phenotype is conserved in our *Drosophila DUX4* model as evidenced by the difficulty in generating transgenic *DUX4* lines and the observed phenotypes. Although clinical symptoms of FSHD manifest in adult skeletal muscles, *DUX4-fl* expression was lethal at the levels dictated by the muscle or ubiquitous GAL4 drivers, and we only obtained viable and fertile flies when expression was restricted to the developing eye. Fortunately this expression pattern still produced a cellular phenotype consistent with FSHD. However, in addition to the eye phenotype, the developmental lethality induced from the ubiquitous *Act5c-GAL4* and *tubP-GAL4* crosses is another screenable phenotype for *DUX4* genetic interactions that may be more relevant in other tissues. Thus, we have generated a viable *Drosophila* model of *DUX4* expression that can be utilized to generate flies that are readily screenable for suppressors or enhancers of the phenotype, the results of which would be of great interest to the FSHD field.

Two myogenic enhancers proximal to the FSHD-associated 4q35 D4Z4 array were recently identified and shown to regulate *DUX4* in differentiated skeletal myocytes [101], providing a potential explanation for the relatively muscle-specific pathology seen in FSHD. In addition, FRG1, a transcriptional target of DUX4-FL in humans, produces a myopathic phenotype generally consistent with FSHD when overexpressed in muscle [36–38]. We have now generated transgenic *Drosophila* lines that, when crossed with an adult muscle-specific GAL4 driver, produce a model of FRG1-mediated muscle dysfunction that recapitulates many aspects of the FSHD phenotype. In addition, FRG1 plays a critical role in both myogenesis and angiogenesis. Crossing to additional GAL4 drivers or performing enhancer/suppressor screens may prove useful in determining the normal biological functions of FRG1 in these tissues, some of which may be relevant to FSHD.

Informative *Drosophila* models have been generated for several neuromuscular diseases and for six of the nine classes of muscular dystrophy including Duchenne [58, 61], myotonic [57, 102], congenital, including certain dystroglycanopathies [58, 59, 103, 104], Emery-Dreyfus [60, 105], oculopharyngeal [56], and limb-girdle [54]; however, no *Drosophila* model for FSHD based on any candidate gene has been reported. Therefore, the *Drosophila* lines presented here that will allow investigations into the functions of two genes important to FSHD and will fill an important void in FSHD research by providing valuable resources for those studying muscle development and FSHD.

## Conclusions

The primary mediator of FSHD pathophysiology is the aberrant stable expression of the DUX4-fl mRNA isoform in adult somatic cells. Expression of DUX4-FL in somatic cells can initiate many potentially adverse downstream events including changes in gene expression, disruption of RNA and protein metabolism, and the induction of apoptosis. However, little is known about the precise pathways affected by DUX4-FL expression and it is still not known which DUX4-mediated changes lead to FSHD pathology. *FRG1* has been proposed to be involved in FSHD, as it is a direct transcriptional target of DUX4-FL, and its overexpression causes a myopathic phenotype in mice through an unknown mechanism. Thus, there is a need in the FSHD field for genetically tractable model organisms to investigate the cellular pathways disrupted by aberrant DUX4-FL and FRG1 expression and to determine which of these pathways are potentially involved in pathogenesis. *FRG1* is very highly conserved between Drosophila and human and readily amenable to model systems approaches. *DUX4*, however, is primate specific and not conserved in *Drosophila*, and thus is reliant on conservation of the relevant cellular machinery and pathways; successful DUX4-based models have been generated in *Xenopus* and Zebrafish despite their lack of DUX4 orthologs. Therefore, we generated transgenic lines of *Drosophila* expressing DUX4-fl or FRG1 under the control of the GAL4-UAS system that produced readily screenable phenotypes consistent with what is known about the functions of these proteins in other systems. Induction of DUX4-fl expression during early development was lethal and expression in the eye imaginal disc produced resulted in white ommatidia and disorganized bristles in the adult eye. Induction of FRG1 in the thoracic muscles leads to a disorganized musculature and impaired flight ability. These *Drosophila* phenotypes will be valuable tools for performing genetic screens to identify components of the DUX4-fl apoptotic pathway and the developmental pathways disrupted by FRG1 expression in muscle. In addition, *Drosophila* models based on human disease genes, including many muscular dystrophies, have been used to discover underlying disease mechanisms and for drug screens to identify potentially therapeutic compounds. The DUX4 and FRG1 transgenic flies reported here will provide the tools for similar investigations into mechanisms of FSHD.

## Supporting Information

S1 Fig Sequence of the codon optimized DUX4-fl open reading frame. The sequence of the DUX4-fl cDNA was codon optimized for expression in *Drosophila melanogaster* and synthesized *in vitro*. Changed nucleotides are indicated in red. * indicates translational stop codon.
(PDF)

S1 Movie Increased FRG1 expression in adult muscle impairs flight (Part 1). Groups of flies were analyzed for the ability to fly. Flies were forcibly dislodged from the surface of a vial and scored for their ability to prevent themselves from falling to the bottom by flying. Those that fell were scored as unable to fly; those that immediately lit upon the sides of the vials did so via flight, thus preventing themselves from falling, and were scored as capable of flying. The adult muscle GAL4 driver strain *DJ667* (left) and the *UAS-DmFRG1* strain (right) were readily able to flying upon being dislodged and return to the sides of the vial. However, the *DJ667 GAL4*, *UAS-DmFRG1* progeny (middle), which produced flies overexpressing DmFRG1 in their adult muscles, were unable to fly and fell to the bottom of the vial when dislodged.
(MOV)

S2 Movie Increased FRG1 expression in adult muscle impairs flight (Part 2). A representative adult *DJ667*,*UAS-DmFRG1* fly, which overexpresses DmFRG1 in its thoracic muscles, appears normal and is capable of walking and flexing its intact wings but is unable to fly. These flies jump into the air for take-off as per usual, however, instead of flying they fall back down immediately.
(MOV)

S1 Table UAS-DUX4 transgenesis results. (PDF)

S2 Table Overexpression of DmFRG1 in adult thorax impairs flight ability. (PDF)

S3 Table Oligonucleotides used for cloning and qPCR. (PDF)
