# Supplementary material for: Transgenic Drosophila for Investigating DUX4 and FRG1, Two Genes Associated with Facioscapulohumeral Muscular Dystrophy (FSHD)
Source: PLoS One. 2016 Mar 4;11(3):e0150938. doi: 10.1371/journal.pone.0150938 (PMC4778869; doi:10.1371/journal.pone.0150938)
Supplement: S1 Fig — The sequence of the DUX4-fl cDNA was codon optimized for expression in Drosophila melanogaster and synthesized in vitro. Changed nucleotides are indicated in red. * indicates translational stop codon. (PDF) [file pone.0150938.s001.pdf]

```
                                HA epitope tag
Translation          M  Y  P  Y  D  V  P  D  Y  A  S  L  A  L  P  T  P  S  D  S  T  L  P
Optimized DUX4-fl    ATGTACCCGTACGATGTGCCAGACTACGCCTCCCTGGCCCTGCCAACCCCAAGCGATTCCACGCTGCCA
DUX4-fl                                                 GCCCTCCCGACACCCTCGGACAGCACCCTCCCC

Translation          A  E  A  R  G  R  G  R  R  R  R  L  V  W  T  P  S  Q  S  E  A  L  R
Optimized DUX4-fl    GCCGAGGCCCGCGGACGCGGACGCCGCCGCCGCCTGGTGTGGACCCCGTCGCAGAGCGAGGCCCTGCGC
DUX4-fl              GCGGAAGCCCGGGGACGAGGACGGCGACGGAGACTCGTTTGGACCCCGAGCCAAAGCGAGGCCCTGCGA

Translation          A  C  F  E  R  N  P  Y  P  G  I  A  T  R  E  R  L  A  Q  A  I  G  I
Optimized DUX4-fl    GCCTGCTTCGAGCGCAACCCATACCCAGGAATCGCCACGCGCGAGCGCCTGGCCCAGGCCATCGGAATT
DUX4-fl              GCCTGCTTTGAGCGGAACCCGTACCCGGGCATCGCCACCAGAGAACGGCTGGCCCAGGCCATCGGCATT

Translation          P  E  P  R  V  Q  I  W  F  Q  N  E  R  S  R  Q  L  R  Q  H  R  R  E
Optimized DUX4-fl    CCAGAGCCACGCGTGCAGATTTGGTTCCAGAATGAGCGCTCCCGCCAGCTGCGCCAGCACCGCCGCGAG
DUX4-fl              CCGGAGCCCAGGGTCCAGATTTGGTTTCAGAATGAGAGGTCACGCCAGCTGAGGCAGCACCGGCGGGAA

Translation          S  R  P  W  P  G  R  R  G  P  P  E  G  R  R  K  R  T  A  V  T  G  S
Optimized DUX4-fl    TCGCGCCCATGGCCAGGACGCCGCGGACCACCCGAGGGACGCCGCAAGCGCACCGCCGTGACGGGCTCG
DUX4-fl              TCTCGGCCCTGGCCCGGGAGACGCGGCCCGCCAGAAGGCCGGCGAAAGCGGACCGCCGTCACCGGATCC

Translation          Q  T  A  L  L  L  R  A  F  E  K  D  R  F  P  G  I  A  A  R  E  E  L
Optimized DUX4-fl    CAGACCGCCCTGCTGCTGCGCGCCTTCGAGAAGGACCGCTTCCCAGGAATCGCCGCCCGCGAGGAGCTG
DUX4-fl              CAGACCGCCCTGCTCCTCCGAGCCTTTGAGAAGGATCGCTTTCCAGGCATCGCCGCCCGGGAGGAGCTG

Translation          A  R  E  T  G  L  P  E  S  R  I  Q  I  W  F  Q  N  R  R  A  R  H  P
Optimized DUX4-fl    GCCCGCGAGACGGGCCTGCCGGAGAGCCGCATCCAGATTTGGTTCCAGAACCGCCGCGCCCGCCACCCA
DUX4-fl              GCCAGAGAGACGGGCCTCCCGGAGTCCAGGATTCAGATCTGGTTTCAGAATCGAAGGGCCAGGCACCCG

Translation          G  Q  G  G  R  A  P  A  Q  A  G  G  L  C  S  A  A  P  G  G  G  H  P
Optimized DUX4-fl    GGACAGGGAGGACGCGCCCCGGCCCAGGCCGGCGGACTGTGCTCGGCCGCCCCCGGCGGAGGCCACCCG
DUX4-fl              GGACAGGGTGGCAGGGCGCCCGCGCAGGCAGGCGGCCTGTGCAGCGCGGCCCCCGGCGGGGGTCACCCT

Translation          A  P  S  W  V  A  F  A  H  T  G  A  W  G  T  G  L  P  A  P  H  V  P
Optimized DUX4-fl    GCCCCAAGCTGGGTGGCCTTCGCCCATACCGGAGCCTGGGGAACGGGCCTGCCCGCCCCGCATGTGCCC
DUX4-fl              GCTCCCTCGTGGGTCGCCTTCGCCCACACCGGCGCGTGGGGAACGGGGCTTCCCGCACCCCACGTGCCC

Translation          C  A  P  G  A  L  P  Q  G  A  F  V  S  Q  A  A  R  A  A  P  A  L  Q
Optimized DUX4-fl    TGCGCCCCCGGGAGCCCTGCCGCAGGGCGCCTTCGTGAGCCAGGCTGCCCGCGCCGCCCCAGCCCTGCAG
DUX4-fl              TGCGCGCCTGGGGCTCTCCCACAGGGGGCTTTCGTGAGCCAGGCAGCGAGGGCCGCCCCCGCGCTGCAG

Translation          P  S  Q  A  A  P  A  E  G  I  S  Q  P  A  P  A  R  G  D  F  A  Y  A
Optimized DUX4-fl    CCCTCGCAGGCCGCCCCCGCCGAGGGAATTAGCCAGCCAGCCCCCGCCCGCGGCGATTTCGCCTACGCC
DUX4-fl              CCCAGCCAGGCCGCGCCGGCAGAGGGGATCTCCCAACCTGCCCCGGCGCGCGGGGATTTCGCCTACGCC

Translation          A  P  A  P  P  D  G  A  L  S  H  P  Q  A  P  R  W  P  P  H  P  G  K
Optimized DUX4-fl    GCCCCGGCCCCCGCCAGACGGAGCCCTGTCCCACCCACAGGCCCCACGCTGGCCCCCGCATCCCGGCAAG
DUX4-fl              GCCCCGGCTCCTCCGGACGGGGCGCTCTCCCACCCTCAGGCTCCTCGCTGGCCTCCGCACCCGGGCAAA

Translation          S  R  E  D  R  D  P  Q  R  D  G  L  P  G  P  C  A  V  A  Q  P  G  P
Optimized DUX4-fl    TCGCGCGAGGATCGCGACCCGCAGCGCGATGGCCTGCCGGGACCATGCGCCGTGGCCCAGCCCGGACCG
DUX4-fl              AGCCGGGAGGACCGGGACCCGCAGCGCGACGGCCTGCCGGGCCCCTGCGCGGTGGCACAGCCTGGGCCC

Translation          A  Q  A  G  P  Q  G  Q  G  V  L  A  P  P  T  S  Q  G  S  P  W  W  G
Optimized DUX4-fl    GCCCAGGCCGGCCCCCAGGGACAGGGCGTGCTGGCCCCACCCACCAGCCAGGGCTCCCCGTGGTGGGGA
DUX4-fl              GCTCAAGCGGGGCCGCAGGGCCAAGGGGTGCTTGCGCCACCCACGTCCCAGGGGAGTCCGTGGTGGGGC
```

```
Translation           W  G  R  G  P  Q  V  A  G  A  A  W  E  P  Q  A  G  A  A  P  P  P  Q
Optimized DUX4-fl   TGGGGCCGCGGACCACAGGTGGCCGGAGCCGCCTGGGAGCCACAGGCCGGAGCCGCCCCACCACCACAG
DUX4-fl             TGGGGCCGGGGTCCCCAGGTCGCCGGGGCGGCGTGGGAACCCCAAGCCGGGGCAGCTCCACCTCCCCAG

Translation           P  A  P  P  D  A  S  A  S  A  R  Q  G  Q  M  Q  G  I  P  A  P  S  Q
Optimized DUX4-fl   CCAGCCCCGCCAGATGCCTCCGCCTCGGCCCGCCAGGGACAGATGCAGGGAATCCCAGCCCCAAGCCAG
DUX4-fl             CCCGCGCCCCCGGACGCCTCCGCCTCCGCGCGGCAGGGGCAGATGCAAGGCATCCCGGCGCCCTCCCAG

Translation           A  L  Q  E  P  A  P  W  S  A  L  P  C  G  L  L  L  D  E  L  L  A  S
Optimized DUX4-fl   GCCCTGCAGGAGCCAGCCCCATGGTCCGCCCTGCCGTGCGGCCTGCTGCTGGACGAGCTGCTGGCCTCC
DUX4-fl             GCGCTCCAGGAGCCGGCGCCCTGGTCTGCACTCCCCTGCGGCCTGCTGCTGGATGAGCTCCTGGCGAGC

Translation           P  E  F  L  Q  Q  A  Q  P  L  L  E  T  E  A  P  G  E  L  E  A  S  E
Optimized DUX4-fl   CCAGAGTTCCTGCAGCAGGCCCAGCCACTGCTGGAGACGGAGGCCCCAGGAGAGCTGGAGGCCAGCGAG
DUX4-fl             CCGGAGTTTCTGCAGCAGGCGCAACCTCTCCTAGAAACGGAGGCCCCGGGGGAGCTGGAGGCCTCGGAA

Translation           E  A  A  S  L  E  A  P  L  S  E  E  E  Y  R  A  L  L  E  E  L  *
Optimized DUX4-fl   GAGGCCGCCTCCCTGGAGGCCCCACTGTCGGAGGAGGAGTACCGCGCCCTGCTGGAGGAGCTGTAA
DUX4-fl             GAGGCCGCCTCGCTGGAAGCACCCCTCAGCGAGGAAGAATACCGGGCTCTGCTGGAGGAGCTTTAG
```

Figure S1: Sequence of the codon optimized DUX4-fl open reading frame. The sequence of the DUX4-fl cDNA was codon optimized for expression in Drosophila melanogaster and synthesized in vitro. Changed nucleotides are indicated in red. * indicates translational stop codon
