# Supplementary material for: Transgenic Drosophila for Investigating DUX4 and FRG1, Two Genes Associated with Facioscapulohumeral Muscular Dystrophy (FSHD)
Source: PLoS One. 2016 Mar 4;11(3):e0150938. doi: 10.1371/journal.pone.0150938 (PMC4778869; doi:10.1371/journal.pone.0150938)
Supplement: S1 Table — (PDF) [file pone.0150938.s004.pdf]

S1 Table: UAS-DUX4 transgenesis results

Constructs*	Larvae	Adult eclosed	Fertile crosses	Transformants	Mortality**	Sterility
UASp-DUX(MKG)	107	96 (89.7%)	86 (89.6%)	24 (27.9%)	0 (0%)	ND
pUAST-DUX(MKG)	90	44 (48.9%)	38 (86.4%)	8 (21.1%)	1 (12.5%)	7 (87.5%)
UASp-DUX(MAL)	95	81 (85.3%)	53 (65.4%)	11 (20.8%)	0 (0%)	ND†
pUAST-DUX(MAL)	81	55 (67.9%)	37 (67.3%)	7 (18.9%)	7 (100%)***	N/A

* Each construct was injected into 125 embryos
** Number of transgenic lines that died before they reproduced. Mortality of each line was confirmed with more than five independent crosses between transgenic flies and white balancer flies.
***Male transgenic flies were very weak and never reproduced. Some of the female transgenic flies were able to reproduce before dying; however, none of them produced transgenic progeny.
† Homozygous animals tend to stop egg production at an early age.
