# Supplementary material for: Transgenic Drosophila for Investigating DUX4 and FRG1, Two Genes Associated with Facioscapulohumeral Muscular Dystrophy (FSHD)
Source: PLoS One. 2016 Mar 4;11(3):e0150938. doi: 10.1371/journal.pone.0150938 (PMC4778869; doi:10.1371/journal.pone.0150938)
Supplement: S2 Table — (PDF) [file pone.0150938.s005.pdf]

S2 Table: Overexpression of DmFRG1 in adult thorax impairs flight ability

Genotypes		n	Impaired Flight Ability
DJ667 GAL4, UAS-DmFRG1 /	Male	160	121 (75.6%)
DJ667 GAL4, UAS-DmFRG1	Female	155	132 (85.2%)
DJ667 GAL4 / DJ667 GAL4	Male	64	9 (14.1%)
	Female	52	7 (13.5%)
