# Supplementary material for: Transgenic Drosophila for Investigating DUX4 and FRG1, Two Genes Associated with Facioscapulohumeral Muscular Dystrophy (FSHD)
Source: PLoS One. 2016 Mar 4;11(3):e0150938. doi: 10.1371/journal.pone.0150938 (PMC4778869; doi:10.1371/journal.pone.0150938)
Supplement: S3 Table — (PDF) [file pone.0150938.s006.pdf]

S3 Table: Oligonucleotides used for cloning and qPCR

DmFRG1 5' *Bam*HI	CCAAGGATCCATCAGACTACGATCATGCACGCATT
DmFRG1 3' *Xba*I	AACCTCTAGATTACTTGCAATACCGATCGGCTTTC
DmFRG1 qPCR For	CATGGACAATGG TCTCTTCACATTG
DmFRG1 qPCR Rev	CATTCGTTGTTC CTCAAAAACCG
rp49 qPCR For	TGCTAAGCTGTCG CACAAATGGC
rp49 qPCR Rev	GCAGTAAACGCGGTTCTGCAT
